# From Heuristic to Mathematical Modeling of Drugs Dissolution Profiles: Application of Artificial Neural Networks and Genetic Programming

**DOI:** 10.1155/2015/863874

**Published:** 2015-05-26

**Authors:** Aleksander Mendyk, Sinan Güres, Renata Jachowicz, Jakub Szlęk, Sebastian Polak, Barbara Wiśniowska, Peter Kleinebudde

**Affiliations:** ^1^Department of Pharmaceutical Technology and Biopharmaceutics, Jagiellonian University Medical College, Medyczna 9 Street, 30-688 Krakow, Poland; ^2^Institute of Pharmaceutics and Biopharmaceutics, Heinrich-Heine University of Düsseldorf, Universitätsstraße 1, 40225 Duesseldorf, Germany; ^3^Department of Pharmacoepidemiology and Pharmacoeconomics and Department of Social Pharmacy, Faculty of Pharmacy, Jagiellonian University Medical College, Medyczna 9 Street, 30-688 Krakow, Poland; ^4^Simcyp (a Certara Company) Limited, Blades Enterprise Centre, John Street, Sheffield S2 4SU, UK

## Abstract

The purpose of this work was to develop a mathematical model of the drug dissolution (*Q*) from the solid lipid extrudates based on the empirical approach. Artificial neural networks (ANNs) and genetic programming (GP) tools were used. Sensitivity analysis of ANNs provided reduction of the original input vector. GP allowed creation of the mathematical equation in two major approaches: (1) direct modeling of *Q* versus extrudate diameter (*d*) and the time variable (*t*) and (2) indirect modeling through Weibull equation. ANNs provided also information about minimum achievable generalization error and the way to enhance the original dataset used for adjustment of the equations' parameters. Two inputs were found important for the drug dissolution: *d* and *t*. The extrudates length (*L*) was found not important. Both GP modeling approaches allowed creation of relatively simple equations with their predictive performance comparable to the ANNs (root mean squared error (RMSE) from 2.19 to 2.33). The direct mode of GP modeling of *Q* versus *d* and *t* resulted in the most robust model. The idea of how to combine ANNs and GP in order to escape ANNs' black-box drawback without losing their superior predictive performance was demonstrated. Open Source software was used to deliver the state-of-the-art models and modeling strategies.

## 1. Introduction

Mathematical description of the process of drug dissolution from the dosage form is a widely discussed and analyzed problem, just to name Higuchi [1] or Korsmeyer and Peppas [2] and more recent works of Siepmann [3, 4]. Modeling based approach allowed classification of controlling mechanism of the drug release process into three main groups: diffusion-, swelling-, and chemically-based, respectively [5]. Such classification is a tool allowing for dissolution rate and mechanism prediction in order to characterize pharmaceutical formulation. However presence of the vast number of excipients, formulation technologies, and manufacturing processes, quantitative characterization of the dissolution process, and its correlation make characterization challenging. The artificial neural networks (ANNs), which are usually computer programs designed to simulate biological neural systems in both their functional activity and structure, are useful data analysis tools to handle such complex, nonlinear relationships. ANNs were utilized in multiple applications in various areas of science and technology [6] along with pharmaceutical sciences, including but not limited to development of nimodipine consisting floating tablets [7], minimisation of the capping tendency during tableting process [8], optimization of galenical dosage form technological process [9], prediction of dissolution from ketoprofen consisting solid dispersions [10], analysis of parameters of direct compression tableting [11], control of quality attributes of tablets manufactured by wet granulation [12], prediction of gentamicin [13], remifentanil [14] and aminoglycosides [15] serum concentrations, analysis of pharmacokinetic population data [16], evaluation of an in vitro-in vivo correlation for nebulizer delivery of salbutamol [17], oral verapamil products [18], sustained release paracetamol matrix tablet formulations [19], and nifedipine osmotic release tablets [20]. However they are also considered the so-called black-box models as it is not possible to provide analytical solution of their way of data processing; what stays in contradiction to the idea that the model purpose is to reveal information about the analyzed system. According to the U.S. Food and Drug Administration's (FDA) Process Analytical Technologies (PAT) initiative, successful modeling is especially important for identification of the mechanisms underlying analyzed process, that is, drug dissolution, which is usually performed via statistical modeling. When there is no a priori knowledge about the analyzed problem such procedure based on finding optimal shape of mathematical function is faced with a problem of unlimited design space without border conditions. This is the obstacle, which ANNs overcome by their ability of self-adaptation to the data. There is however a gap between statistical modeling and ANNs which does not allow the advantages of both approaches to explore simultaneously without encountering their drawbacks. Fuzzy logic and so-called neurofuzzy systems (NFs), where the ANN's structure is encoded in the table of the logical rules [21, 22], are an attempt to overcome this obstacle. The logical rules of NFs are easy to follow, provided that they were built with a reasonably small number of rules. Yet the predictive performance of NFs is sometimes below the level achieved by multilayered perceptron artificial neural networks (MLP-ANNs), due to the structure of NFs designating them mostly to the classification problems. A possible solution might be the use of genetic programming (GP), a tool belonging to the computational intelligence empirical modeling systems, founded on the ground of evolutionary computation and able to automatically generate mathematical equations, based on the data [23–25]. The most important GP drawbacks are large computational power demand and tendency to create very complex equations which can be controlled by a priori limited length of the chromosome; however this parameter is to be adjusted by trial and error.

This work objective was to show how to use ANNs and GP cooperatively in order to create effectively a simple mathematical model of the drug dissolution from the dosage form, based on the empirical approach.

## 2. Materials and Methods

### 2.1. Dataset

The dataset contains results of dissolution tests carried out for 5 various formulations of lipid extrudates. Extrudates consisting of diprophylline (Diprophylline Base, BASF, Ludwigshafen, Germany), tristearin (Dynasan 118, Sasol, Witten, Germany), and polyethylene glycol of a mean molecular weight of 20000 (Polyglykol 20000, Clariant, Sulzbach, Germany) in a 50 : 45 : 5%-ratio (w/w/w) were produced by solid lipid extrusion [26]. Extrudates were varied by their length (*L*) and diameter (*d*), where *L* was in the range from 5 mm to 18 mm and *d* between 0.6 mm and 3.5 mm (Table 1). Both *L* and *d* were established as time-independent variables characterizing particular formulation.

Dissolution experiments were carried out according to the USP 32 method 1 up to the 1000 minutes with sampling interval of 5 minutes. All the assays were run in triplicate. Totally, the database consisted of 1000 records of the data. There were originally 3 inputs of the system: the length (*L*) and the diameter (*d*) of the extrudate and the dissolution time (*t*). The output (*Q*) was the amount (%) of diprophylline released at the time (*t*) specified at the input. The dataset was preprocessed in several ways:

- noise addition to prevent models from overfitting: the noised data records were generated numerically from the continuous distribution within range of ±5% amplitude of the each variable value;
- data records balancing according to the output minimum/maximum values ratio: the number of the data records was multiplied by increasing the number of data records with smaller values of the output variable;
- linear scaling using either output range of 〈0.2,0.8〉 or 〈−0.8,0.8〉, in order to match nonlinear activation functions domains, was used for ANNs only.

### 2.2. Modeling

**Figure 1:**
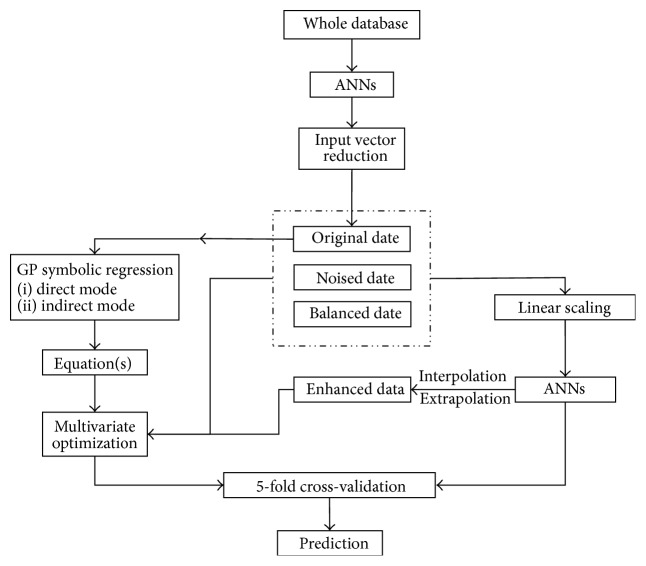
Workflow diagram presenting modeling methodology.

Modeling was carried out with the use of computational intelligence tools, namely, MLP-ANNs and GP, along with nonlinear regression tools. The whole procedure was carried out in the following steps (Figure 1):

- ANNs modeling to reduce the input vector and to create black-box predictive models,
- GP modeling based on the reduced input vector to create mathematical equations,
- multivariate optimization to fit parameters of the equations to the available data.

The following activation functions were tested for MLP-ANNs: linear, logistic, hyperbolic tangent, and logarithmic function (fsr) and their architectures consisted of 1 to 7 hidden layers. In addition to the MLP-ANNs, NFs of the simplest Mamdani type were employed [22] and contained from 5 to 100 nodes in the hidden layer. All models were multiple-input-single-output (MISO) type and the backpropagation training algorithm had few modifications:

- *momentum* technique with the factor 0.3;
- *delta-bar-delta* algorithm with the initial learning factor 0.65;
- *jog-of-weights* technique designed to prevent of getting stuck in the local minima of the cost function; a simple noise addition to the weights while the ANN was not improving its efficiency during the 100,000 epochs (the patience criterion).

The 5-fold cross-validation applied during the study is a derivative of the bootstrap technique. It means that each model was trained 5 times, each time on different dataset. The procedure of the datasets preparation is based on a simple data records withdrawal with replacement. The amount of the withdrawn data was 20% of the original database each time and it resulted in the testing dataset accompanied by the remaining 80% of the data consisting the training dataset. Once withdrawn, the data record could not be used for the testing dataset anymore. Thus, at the end of the process there were 5 pairs of the training-testing datasets created. In this work an enhanced scheme of the data partitioning was employed, where each time all the data belonging to the particular formulation were excluded, thus simulating the real application of the model forced to predict behavior of the unknown formulation. 5-fold cross-validation was employed, and not the usually applied 10-fold cross-validation scheme, as there were 5 variants of the dimensions of the extrudates present in the database.

Totally, there were 208 types of ANNs models developed, where the type of the model differed from all others for

- architecture of ANN (MLP-ANNs and NFs),
- training dataset modifications.

Considering 5-fold cross-validation, it added up to 1040 models trained in the single run. Each model was trained up to 1,000,000 epochs with several stop points, where the generalization error was assessed in order to find the most optimal training conditions. The stop-points were as follows: 5 000, 10 000, 15 000, 20 000, 30 000, 50 000, 100 000, 150 000, 200 000, 300 000, 500 000, 750 000, and 1 000 000 epochs. The epoch size was equal to 1. Sensitivity analysis was used for reduction of the inputs number. It was carried out according to the Żurada's method [28] with further modifications by Mendyk [10, 29]. The latter included use of collective results from the set of the best trained ANNs instead of the single ANN model like in the original approach.

Mathematical equations were generated by means of GP and the symbolic regression mode available in the* rgp* package [30] of the Open Source statistical environment *R* [31]. Two modeling approaches were applied:

- direct mapping of the input versus output variables;
- indirect mapping through arbitrary preselected mathematical equation.

The direct mapping means that GP was working with the original dataset, attempting to create functional relationship between the amount (%) of the drug released (*Q*) and the vector of parameters containing the time variable (*t*) accompanied by geometric characteristics (*θ*) of the formulation:

(1) $$\begin{array}{c} Q=f\left(\;\theta ,t\;\right), \end{array}$$

where *Q* is the amount of the drug released (%) at *t* time and *θ* is a vector of the parameters characterizing formulation (diameter and length of the extrudates).

Indirect mapping was carried out with the use of Weibull equation:

(2) $$\begin{array}{c} Q=100\cdot \left(\;1-\exp\left(\;\frac{-t^{A}}{K}\;\right)\;\right), \end{array}$$

where *Q* is the amount of the drug released (%) at *t* time and *A*, *K* are Weibull equation constants.

In the first step, the profiles were fitted to Weibull model. The second stage involved GP to find the relationships between Weibull's constants relevant to the dissolution profiles and the geometric characteristics of the respective formulations (3) and (4). New datasets were created: one for *A* versus *θ* (3) and the second for *K* versus *θ* (4). Consider

(3) Af1θ,

(4) K=f2θ,

where *A*, *K* are Weibull equation constants and *θ* is a vector characterizing geometry of the formulation.

Both datasets contained 5 data records as each dissolution profile was characterized by single set of the Weibull *A* and *K* constants.

In order to ensure mathematical models simplicity, GP runs were carried out with the solution complexity control, where the maximum tree depth (the chromosome length) was minimized with regard to the prediction error yielded by the model. Thus, the parameter “*individualSizeLimit*” of the* rgp* package was varied from 10 to 300 and was subject of the optimization with regard to the goodness of fit expressed as the root mean squared error (RMSE):

(5) $$\begin{array}{c} \text{RMSE}=\sqrt{\frac{\sum_{i=1}^{n}{\left({\text{pred}}_{i}-{\text{obs}}_{i}\right)}^{2}}{n}}, \end{array}$$

where obs_*i*_ and pred_*i*_ are the observed and predicted values respectively, *i* is the data record number, and *n* is the total number of records.

In order to ensure variability of the population, the number of subjects in the population (*populationSize*) was set to 10,000. Other parameters of* rgp* were as follows:

- *myfunctionSet*, a set of prototype functions, was restricted to the simplest arithmetic operators like addition, subtraction, multiplication, and division together with power, natural logarithm, square root, and exponent function;
- the algorithm stop condition* makeFitnessStopCondition* (RMSE) was equal to 5.0 and 0.01 for *K* and *A* Weibull's constants, respectively, but for the direct GP modeling it was set to 1.0;
- *makeTimeStopCondition* was set to 1 hour for indirect and 120 hours for direct modeling mode.

Both of the above mentioned modes of GP runs were performed on the whole original datasets without any modifications or preprocessing. As GP provides the model with optimized values of its parameters, the final stage of the modeling was multivariate optimization used to fit parameters of the created equations* de novo*, starting from the constant values (0.1). Fitting was performed either for the whole database or in the mode of 5-fold cross-validation. Nonlinear optimization was performed by multistage approach programmed in the *R* script. Two *R* packages were employed in the following sequence:

- *optim* with* SANN* (simulated annealing) optimization method;
- *optimx* with directives:* all.methods* =* TRUE* and* follow.on* =* FALSE* [32].

The rationale behind such complicated procedure was to find the best possible model starting from the random values of parameters. The first stage of the* optim* method was the simulated annealing algorithm, a global optimization nongradient method. It was used to provide better start-point to the following* optimx* procedure, which was carried out with all the algorithms available (*all.methods* =* TRUE*). However,* optimx* was run not in the sequence but separately (*follow.on* =* FALSE*), each optimization method starting from the parameters set previously by* SANN* method. At the end, the best model's parameters were selected and the resulting model was tested on the separate dataset (the testing dataset) to assess its generalization ability. The parameters' selection criterion was the model goodness of fit expressed as the RMSE obtained on the training dataset. Noise addition to the data and its balancing were employed in the same manner as for ANNs based approach. Additionally, ANNs were also used for the data preprocessing. The new (enhanced) datasets were created by interpolation and extrapolation of the original training datasets used in the 5-fold cross-validation procedure (Figure 1). Sampling of the design space was carried out with a step of 3.33% of the minimum-maximum range of the variable. The boundaries were set between minimum and 110% of the maximum variable value, thus allowing for the extrapolation of the original dataset. If, by any chance, the artificial training data records overlapped with the respective test dataset, they were discarded, thus ensuring pure generalization assessment. The original datasets were merged with the artificially created data. The final sizes of the enhanced training datasets were between 7400 and 7800 records.

The predicted and observed dissolution profiles were compared using similarity factor, *f*_2_, computed according to the FDA:

(6) $$\begin{array}{c} f_{2}=50\cdot \log\left\{\;{\left[\;1+\left(\frac{1}{n}\right)\cdot \overset{n}{\underset{t=1}{\sum }}{\left(R_{t}-T_{t}\right)}^{2}\;\right]}^{-0.5}\cdot 100\;\right\}, \end{array}$$

where *n* is the number of time points, *R* is the dissolution value (%) of the prechange batch at time *t*, and *T* is the dissolution value (%) of the postchange batch at time *t*.

All computations were performed on 21 PC workstations organized into the grid structure and working under Linux operating systems control. An own-written ANNs simulator* Nets2012* [10, 29, 33] was used in this study for neural modeling.* KinetDS 3.0* software was used for fitting the dissolution profiles [34].

## 3. Results and Discussion

### 3.1. Results

The first stage of the research was neural modeling, where ANNs were trained to create black-box models connecting dissolution profile with the time (*t*), the diameter (*d*), and the length (*L*) variables. As a result of the sensitivity analysis procedure, ANNs selected two the most important inputs: the time variable and the diameter of the extrudate.

The best ANN yielded generalization error RMSE = 2.18. For the comparison, there were additionally two types of ANNs models trained: (1) based on the three original inputs and (2) based on the two inputs including the sampling time (*t*) and the length of the extrudate (*L*). The above variants of input vectors were prepared to challenge results of sensitivity analysis with ultimate objective of the best possible predictability of the model achieved with minimum number of variables. In all cases the same conditions of 5-fold cross-validation were retained. A comparison of the generalization errors of the above mentioned three types of models supports sensitivity analysis choice of the crucial variables, where the length of the extrudate (*L*) was found to be not important for the drug release profile (Table 2).

**Figure 2:**
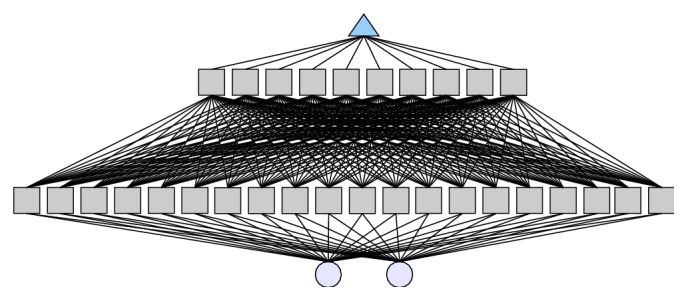
Optimal ANN architecture containing 20 and 10 nodes in the hidden layer and the 2 elements based input vector: the diameter of the extrudate (*d*) and the sampling time (*t*).

Regarding the two elements input vector (*d* and *t*), ANNs model optimization based on the 5-fold cross-validation procedure was performed, where the best architecture was chosen based on the criterion of its generalization ability. The best neural model found consisted of two hidden layers with 20 and 10 nodes, respectively, and hyperbolic tangent activation function (Figure 2). The training iterations number was 1,000,000 and the training dataset was noised and scaled into the range of 〈−0.8,0.8〉.

Both GP runs, the direct and indirect one, were successful in terms of finding representative model. This means that the system was able to assign mathematical formulas to the relationships presented as the modeling goals. Based on the selection criteria, the following mathematical models were chosen (7)–(10). One has

(7) $$\begin{array}{c} Q=\frac{\left(\ln\left(d\right)+d+c_{1}\right)\cdot \left(t+c_{2}\right)}{t-\sqrt{t}+c_{3}\cdot d^{2}}+d^{2}, \end{array}$$

where *Q* is the amount of drug released (%) in time *t*, *d* is the extrudate diameter, and *c*_1–3_ are adjustable parameters.

In case of the direct GP modeling (7) was selected as the best model. It was relatively simple, with three parameters (*c*_1_, *c*_2_, and *c*_3_) and contained no pyramids of the powers like that in (9).

The indirect GP modeling resulted in the two equations describing relationships between the constants of the Weibull equation and the diameter of the extrudates:

(8) Kc1·dd+c2·d,A=c3d,

where *A*, *K* are Weibull equation constants, *c*_1–3_ are adjustable parameters, and *d* is the diameter of the extrudate.

Substituting constants of the original Weibull equation with (8) resulted in the final model relating directly the release of the drug substance to the time variable and the extrudate diameter:

(9) $$\begin{array}{c} Q=100\cdot \left(\;1-\exp\left(\;\frac{-t^{c_{3}^{\sqrt{d}}}}{c_{1}\cdot d^{d}+c_{2}\cdot d}\;\right)\;\right), \end{array}$$

where *Q* is the amount of drug released (%) in time *t*, *d* is the extrudate diameter, and *c*_1–3_ are adjustable parameters.

The above equations are even simpler than previously reported (10) [27]. This is the result of more thorough investigation of the GP stopping conditions and also longer run times extended up to 120 hours per CPU core

(10) Q=100·1−exp⁡−tc5d+c6exp⁡c1·d+c2·dln⁡c3·d+c4,

where *Q* is the amount of drug released (%) in time *t*, *d* is the extrudate diameter, and *c*_1–5_ are adjustable parameters.

Equations (7), (9), and (10) were fitted to the various datasets: the original one and its preprocessed derivatives (noised, balanced, and enhanced with ANN model predictions). The models fitting procedure resulted in the generalization errors estimation (Table 3), which accounted for each model's robustness. It is clearly seen that (7) is the most robust as its predictive performance is not impaired by preprocessing of the training dataset. The above mentioned fitting procedure followed the 5-fold cross-validation scheme; therefore it might be directly compared to the results of the ANNs modeling (Table 2). It was noted that mathematical equations are comparable to the ANNs in their predictive performance. Another performance verification of (7) was based on the mechanistic modeling presented by Güres et al. [27], where similarity factors (*f*_2_) were computed for the simulated and observed data (Table 4).

**Figure 3:**
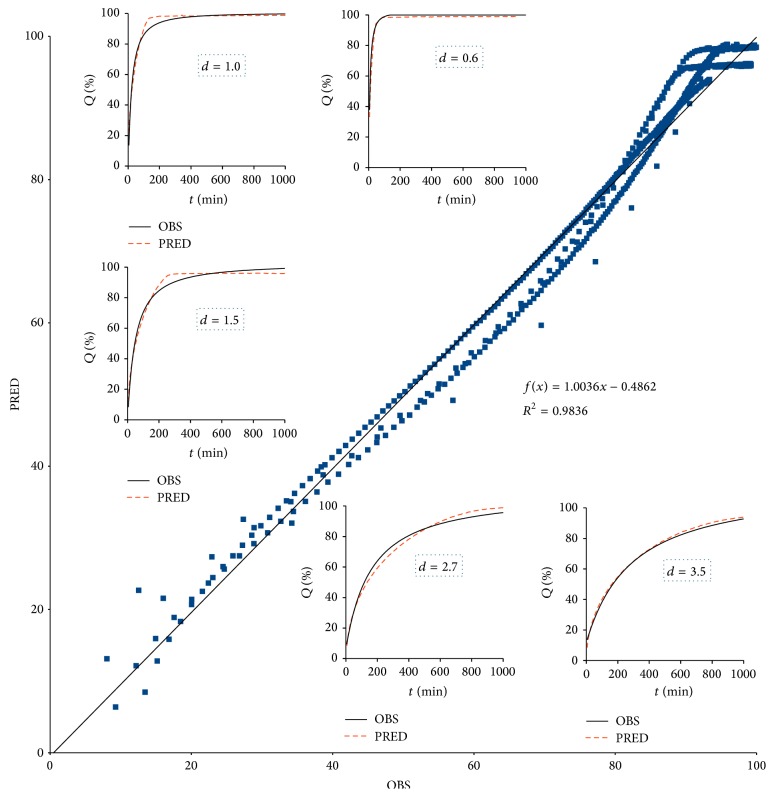
Generalization results for (7).

Graphical representation of the generalization results of (7) were presented as Figure 3, where overall relationship of predicted versus observed accounts for all 5-folds of cross-validation gathered together. Another illustration of (7) robustness was depicted in Table 5, where parameters of (7) resulted from the fitting procedure performed by means of the 5-fold cross-validation.

### 3.2. Discussion

According to Figure 1, there were two main branches of the conducted research: ANNs and GP based approach. They were applied simultaneously yet not completely separately. Following Figure 1 it might be noticed that ANNs and GP were building the models based on the same datasets and procedures (5-fold cross-validation), with three distinct exceptions:

1. input vector reduction was performed by ANNs only;
2. GP runs were enhanced by the knowledge about the problem, namely, Weibull equation used as a framework for the mathematical equations development;
3. mathematical models created by GP were improved by the use of ANNs-enhanced datasets.

The procedure of the input vector reduction was carried out with ANNs due to their effectiveness in this task. Although, GP is capable of the crucial variables selection by pure empirical approach, it would take very long time to optimize mathematical function shape, number of its parameters, and independent variables simultaneously. Therefore, use of ANNs was recognized here as a simple management of computational resources. ANNs provided not only the means of the crucial variables selection but also justification of the claim that for this problem the diameter (*d*) and the sampling time (*t*) variables are the crucial ones. Based on the estimation of generalization abilities of ANNs models trained on the datasets with different input vectors, it could be concluded that the extrudate length (*L*) variable is not important. The *L* is not improving the predictive power of ANNs based on the 3-element input vector and is certainly not enough as a single variable along with the *t* in the models with the 2-element input vector (Table 1).

Once the input vector was established, the modeling was conducted both ways: with the use of ANNs and GP. It is noteworthy that predictive modeling with ANNs was also the element of the validation and justification of the input vector reduction procedure. It is another point to the discussion about the cost-effectiveness of the neural modeling. In case of GP runs, the indirect mode was originally meant to be primary as it was assumed that inclusion of the external knowledge about the problem should be beneficial to the model. Indeed, it was possible to find several well-predicting models with Weibull equation used as a framework. Choice of Weibull model requires clarification. It was mainly due to its flexibility and ability to create good representation of the dissolution profiles. However, more deep rationale was of methodological nature, as this work was dedicated to the empirical modeling, where the Weibull model is a good example, thus not introducing any* a priori* knowledge into the presented results. The simplest model found in the indirect modeling procedure was presented in (9). It contains only 3 adjustable parameters (*c*_1_, *c*_2_, and *c*_3_); thus it is not much more complex than the original Weibull equation and certainly less complex than previously reported (10) [27]. The direct GP run was meant to be the control for the indirect mode and yet, unexpectedly, it produced a very compact equation (7) with good predictive abilities (Table 3 versus Table 2) and again with only three adjustable parameters. This outcome required further investigation. In order to compare (7) with (9), both equations' parameters were optimized on the differently preprocessed datasets resulting in different models but tested according to the 5-fold cross-validation on the same sets of testing data. The results summarized in Table 3 revealed poor stability of (9) demonstrated in the large differences between generalization errors achieved by models optimized on different datasets. On the contrary, satisfactory robustness against the modified datasets was observed for (7), which was the final indication for the best model choice.

ANNs played an important role in this stage of the work. The enhanced dataset was used for the optimization of the parameters of (7) and (9) among the other preprocessed datasets (Figure 1). It was nothing else but the response surface of the best ANNs model with regard to the artificial input data created by the deliberate sampling of the experimental design space (plus limited extrapolation). Use of ANNs-enhanced datasets was meant to provide mathematical equations with all the knowledge of the ANNs. This was clearly beneficial to the less robust (9), which achieved predictive abilities at the level of ANNs only for this type of the data preprocessing (Table 3). Equation (7) was also compared in its predictive abilities to the previously derived mechanistic equation [27] by means of similarity factor (*f*_2_) computed for the modeled versus observed results (Table 4). The results of this comparison favor (7) over mechanistic model and are in the same time in accordance with the conclusions presented by Güres et al. [27], namely, the low importance of the extrudates length parameter (*L*). Another confirmation of (7) robustness was delivered by comparison of the values of its parameters fitted during the 5-fold cross-validation. It is noteworthy that the value of the coefficient of variation (CV) exceeded 5% only in case of the parameter *c*_2_ (Table 5). It is also an indication for the importance of this parameter. Judging by the position of *c*_2_ in (7) it might be hypothesized that it is associated with the time variable and thus might be directly associated with the drug release kinetics. This hypothesis would require further insight in the future.

It is also worth noting that all the above presented results were obtained with the use of the Open Source software, namely, *R* statistical package. The ANNs simulator* Nets2012*, although not released as Open Source, was own-written with use of* Lazarus*, the Open Source Rapid Application Development (RAD) for the* freepascal* language [35]. The same applies to the* KinetDS* software which was released under GPLv3 license [34]. This study is an example of how Open Source might be exploited to create sophisticated models and modeling strategies. Use of these tools is worth of consideration in order to provide reliable and reproducible solutions at low cost of their development.

## 4. Conclusions

By careful combination of two major heuristic techniques, namely, ANNs and GP, it was possible to build a classical mathematical model of the drug release from the solid lipid extrudates. It is a proof of concept that careful use of both tools might be beneficial to the final model structure and its performance.

GP was enhanced by ANNs in three different ways:

1. sensitivity analysis of ANNs models allowed reduction of the input vector;
2. ANN-enhanced datasets improved predictive performance of some classical models;
3. ANNs modeling results provided knowledge about achievable models' predictive performance for this particular problem, thus creating an endpoint for the mathematical modeling.

The above mentioned ANNs functions make them valuable tools for modeling in pharmaceutical sciences despite their “black-box” nature. Moreover, presented methodology based on the machine learning tools introduces good data processing abilities to the mathematical modeling, which might be of help in the future development of the mechanistic models of drug dissolution, when more physical factors would be taken into the consideration.

## Figures and Tables

**Table 1:** Dimensions of the extrudates: *d*: diameter, *L*: length.

*d* [mm]	*L* [mm]
0.6	14
1.0	10
1.5	29
2.7	8
3.5	5

**Table 2:** Best results of the 5-fold cross-validation procedure conducted for various input vectors based ANNs models.

Input vector	RMSE
2 inputs: diameter (*d*) and time (*t*)	2.18
2 inputs: length (*L*) and time (*t*)	8.58
3 inputs: diameter (*d*), length (*L*), and time (*t*)	2.28

**Table 3:** Generalization errors (RMSE) obtained in the 5-fold cross-validation procedure for three equations fitted to the various datasets.

Dataset	Equation (7)	Equation (9)	Equation (10)
Original	2.32	21.02	10.58
Balanced	2.45	37.98	5.32
Noised	2.29	24.17	5.36
ANN-enhanced	2.33	2.19	2.13

**Table 4:** Similarity factor (*f*_2_) of predicted versus observed curves for (7) and previously derived equation by Güres et al. [27].

Extrudate diameter [mm]	Equation (7)	Güres et al. [27]
0.6	53.0	51.7
1	70.3	50.7
1.5	76.7	56.0
2.7	73.7	57.2
3.5	90.0	67.6

**Table 5:** Comparison of the parameters of (7) across the 5-fold cross-validation.

Number	*c*_1_	*c*_2_	*c*_3_
1	97.76	−1.08	23.81
2	97.25	−1.79	23.26
3	97.05	−1.96	23.08
4	96.72	−1.73	22.40
5	97.14	−1.62	23.36
**CV**	**0.39**%	**20.42**%	**2.21**%
